# Psychometric properties of the Adverse Childhood Experiences Questionnaire 10 item version (ACE-10) among Hungarian adolescents

**DOI:** 10.3389/fpsyg.2023.1161620

**Published:** 2023-05-19

**Authors:** Beáta Kovács-Tóth, Barnabás Oláh, Ildikó Kuritárné Szabó, Zita Fekete

**Affiliations:** ^1^Department of Behavioural Sciences, Faculty of Medicine, University of Debrecen, Debrecen, Hungary; ^2^Doctoral School of Health Sciences, University of Debrecen, Debrecen, Hungary

**Keywords:** adolescents, adverse childhood experiences, measures, psychometrics, validation

## Abstract

**Introduction:**

Although a number of studies have been conducted since the 1995 initiation of the ACE study to map the effects of adverse childhood experiences, few studies have examined the psychometric properties of the individual versions of the ACE questionnaire.

**Aims:**

The Adverse Childhood Experiences Questionnaire 10 item version (ACE-10) has only been tested in a single study in an adult population, while its applicability in a particularly vulnerable population, the adolescents, has not been investigated yet. Our present study aims to address this gap in an adolescent sample of 792 subjects from a non-representative general population.

**Methods:**

Besides demographic data, the Adverse Childhood Experiences Questionnaire 10 item version (ACE-10), the Strengths and Difficulties Questionnaire (SDQ), and the HBSC Symptom Checklist (HBSC-SCL) were employed.

**Results:**

Our results showed acceptable internal consistency (ɵ = 0.86, α = 0.64) and adequate internal validity (r = 0.28–0.70, p < 0.001). In addition, proper concurrent criterion validity of the questionnaire was found when tested along the SDQ and HBSC-SCL items.

**Conclusion:**

Our results demonstrate that the ACE-10 is suitable for assessing intrafamilial adverse childhood experiences in adolescents.

## 1. Introduction

Estimates suggest that up to 1 billion children worldwide may be affected by abuse, which means that every other 2–17-year-old child will experience some form of abuse every year (Hillis et al., 2016). Every third child is affected by emotional abuse, and every fourth child worldwide lives with a mother who is the victim of domestic violence (Stoltenborgh et al., 2012; Hillis et al., 2016; UNICEF, 2017). Moreover, about 120 million girls in the world are the victims of some form of sexual violence before the age of 20 (UNICEF, 2014; UNESCO, 2019).

The Adverse Childhood Experiences Study (based on the concept of trauma including abuse and neglect, later also household dysfunction) (hereinafter: ACE study), was initiated in 1995 in the USA, and aimed to examine the effect of adverse experiences one had in their childhood (before the age of 18) on their adult health. This research, including 17,000 participants, has been going on for over 25 years; its results show that prolonged and accumulated adverse childhood experiences (abuse, neglect, household dysfunction) contribute to the development of various mental and somatic disorders that span into or develop in adulthood (Felitti et al., 1998; Hughes et al., 2017; Bellis et al., 2019).

Several studies in children and adolescents—although fewer in number than in adults—have looked at different aspects of exposure to adverse experiences. These studies found that exposure to adverse childhood experiences among young children and adolescents is associated with health complaints (Flaherty et al., 2006; Kovacs-Toth et al., 2021), poor somatic health status and certain somatic diseases, eg., obesity, bronchial asthma (Boynton-Jarrett et al., 2008; Pretty et al., 2013; Wing et al., 2015; Schroeder et al., 2021), health risk behavior (Hillis et al., 2004; Bomysoad and Francis, 2020), impairment of cognitive functions (Spann et al., 2012; Lum et al., 2015); externalizing behaviors (Lansford et al., 2002; Schilling et al., 2007), internalizing symptoms (Heneghan et al., 2013; Suzuki and Tomoda, 2015), and attachment disorders (Finzi et al., 2001; Cyr et al., 2010).

Data on prevalence are needed to identify the children exposed to violence in a society, and monitor trends in exposure (Sethi et al., 2013). Unfortunately, there are only a few screening tools for the retrospective assessment of traumatic experiences suffered in childhood and adolescence.

One of the most commonly used instruments is the Adverse Childhood Experiences (ACE) Questionnaire (Felitti et al., 1998). The first ACE Questionnaire assesses physical, emotional, sexual abuse and household dysfunction with 17 questions. Household dysfunction includes alcohol or drug use, mental illness in the family, mother treated violently and criminal behavior in household. This first version of the ACE questionnaire excludes questions referring to emotional and physical neglect, but some of the later versions already include them (Felitti et al., 1998; Dube et al., 2004).

Even though it is a commonly used tool, only a few studies have been performed into the psychometric properties of the different types of ACE questionnaires. One such study investigated the psychometric properties of a shortened version of the ACE (Ford et al., 2014), which contains 11 items assessing exposure to nine types of ACEs: verbal, physical, and sexual abuse, household mental illness, household alcohol abuse, household drug abuse, domestic violence, parental separation/divorce, and incarcerated family members. This version of the ACE questionnaire also excludes questions referring to emotional and physical neglect. The psychometric testing of this 11-item version found a three-factor structure: physical/emotional abuse, household dysfunction, and sexual victimization, and showed a good construct validity and adequate internal consistency.

The Adverse Childhood Experiences Abuse Short Form (ACE-ASF), an 8-item retrospective self-report questionnaire measuring lifetime physical, emotional, and sexual abuse, was developed in 2012 by the World Health Organization (WHO). This questionnaire does not measure household dysfunctions; still, results regard it as a valid measure of physical, emotional, and sexual abuse in school-aged adolescents (Meinck et al., 2017).

The short, 10-item screening version of the ACE (ACE-10) assesses the exposure to 10 types of ACEs (emotional, physical and sexual abuse, emotional and physical neglect, and five household dysfunctions: parental separation/divorce, household physical violence, household substance abuse, household mental illness or suicide attempt, incarcerated household member) with 10 single-item questions. In this questionnaire one type of ACEs examined one single question. To the best of our knowledge, only one study so far has investigated its psychometric properties (Wingenfeld et al., 2011); nevertheless, it found good internal consistency and construct validity in the 10-item version. In additions, high correlations with childhood trauma inventories (CTQ—Childhood Trauma Questionnaire) were confirmed. Unfortunately, no information is available regarding the other psychometric properties of this screening tool.

The aim of our current study was to assess the psychometric properties of the Adverse Childhood Experiences Questionnaire 10 item version (ACE-10), and to demonstrate its reliability and validity in a sample of Hungarian adolescents from a non-representative general population. The first originality of our study is that we are investigating an adolescent sample. Another novelty of our paper is that we provide comprehensive information on the psychometric properties of the ACE-10, as the psychometric properties of this screening tool are less comprehensively represented in the previous single study.

## 2. Methods

### 2.1. Sampling and data collection

Our study was a cross-sectional retrospective study. Data collection was conducted between March 2019 and January 2022 in a general community sample of Hungarian adolescents. Altogether 14 schools from 9 settlements were contacted, where we visited students from grade 6 to 11, whose age ranged from 12 to 17 years. Our choice of schools was in line with the type of the settlement so that we could have both village schools and various town or city schools represented in our sample. After parents signed the parental informed consent, the adolescents participating in the study also gave their written informed consent to take part in the study. Students filled in the questionnaires anonymously. Data collection was performed in groups, under the assistance of health psychology master students.

Altogether 907 adolescents were contacted. Parental informed consent rate was 99.4%. Due to student absenteeism on the days of data collection the final response rate was 87.3%. Accordingly, our analysis was carried out on a sample of 792 adolescents. In the first step, the psychometric properties and reliability of the Adverse Childhood Experiences Questionnaire 10 item version (ACE-10) were computed on our complete sample of 792 adolescents (hereinafter: total sample).

However, as statistical analyses only worked in complete datasets, we had to limit criterion validity analysis to a restricted sample. Therefore, in the second stage of the analysis participants with missing data were removed from the sample. This resulted in a subsample of 677 adolescents (hereinafter: subsample). To assess the concurrent criterion validity of the measure, two additional criterion questionnaires were administered, and statistical analyses were controlled for potential confounders such as gender, age, location, and maternal education.

Ethics approval was issued by the Research Ethics Committee of the Hungarian Medical Research Council under the approval number ETT TUKEB 47848–7/2018/EKU. The demographic features of the sample are provided in Table 1.

**Table 1:** Demographic and ACE characteristics of the samples.

	Total sample *n* = 792	Subsample *n* = 677
Gender n (%)		
Boys	322 (40.65)	262 (38.70)
Girls	470 (59.34)	415 (61.29)
Age mean (SD)	14.98 (1.19)	14.95 (1.76)
Location n (%)		
Village	207 (26.13)	173 (25.55)
Town	357 (45.07)	294 (43.42)
City	228 (28.78)	210 (31.02)
Maternal education n (%)^i^		
Primary or lower than primary	73 (10.09)	63 (9.30)
Secondary	264 (36.51)	249 (36.78)
Tertiary	386 (53.38)	365 (53.91)
ACE score n (%)^i^		
0	418 (52.77)	372 (54.94)
1	172 (21.71)	149 (22.01)
2	88 (11.11)	83 (12.25)
3	37 (4.67)	31 (4.57)
≥4	49 (6.18)	42 (6.20)
ACE categories exposure n (%)		
Emotional abuse	106 (13.38)	92 (13.58)
Physical abuse	54 (6.81)	44 (6.49)
Sexual abuse	29 (3.66)	24 (3.54)
Emotional neglect	108 (13.63)	95 (14.03)
Physical neglect	22 (2.77)	15 (2.21)
Parental separation/divorce	196 (24.74)	173 (25.55)
Household physical violence	28 (3.53)	22 (3.24)
Household substance abuse	66 (8.33)	55 (8.12)
Household mental illness	61 (7.70)	53 (7.82)
Incarcerated household member	41 (5.17)	35 (5.16)

^i^The category in the total sample does not add up to the full sample size due to some missing data.

Total sample = ACE Score Calculation properties and reliability analyses; Subsample = ACE Score Calculation concurrent validity tests with SEB symptoms and SHCs; no statistically significant differences between samples in demographic and ACE characteristics (Pearson’s chi-squared test)

### 2.2. Measures

The data were collected using a self-report questionnaire battery. Besides demographic data (gender, age, location, parental educational attainment) and the Adverse Childhood Experiences Questionnaire 10 item version (ACE-10), the Strengths and Difficulties Questionnaire (SDQ) (Goodman et al., 1998; Turi et al., 2011), and the Health Behavior of School Children Symptom Checklist (HBSC-SCL) (Németh and Költő, 2014; Inchley et al., 2016) were also employed to assess concurrent criterion validity. The reason for our choice was that multiple evidences confirmed that the constructs measured with the SDQ and HBSC-SCL are significant correlates of the cumulative ACE score (Flaherty et al., 2006; Hughes et al., 2017; Kovacs-Toth et al., 2021).

#### 2.2.1. The Adverse Childhood Experiences Questionnaire 10 item version

The Adverse Childhood Experiences Questionnaire 10 item version (ACE-10) is a self-report retrospective questionnaire consisting of 10 items (Anda et al., 2010). The first adult version of the ACE-10 had been translated into Hungarian by Ujhelyiné Nagy et al. (2019). However psychometric analyses of the test have not been conducted so far.

The questionnaire assessing exposure to 10 types of intrafamilial ACEs belonging to two categories: five types of maltreatment (1. physical abuse, 2. emotional abuse, 3. sexual abuse, 4. physical and 5. emotional neglect), and five types of household dysfunction (6. parental separation/divorce, 7. household physical violence, 8. household substance abuse, 9. household mental illness or suicide attempt, 10. incarcerated household member). The questionnaire focused on 10 questions requiring a yes/no answer. To reduce subjectivity of perception, the survey investigates common and/or severe behavioral patterns by providing concrete examples (e.g., for emotional abuse: “Did a parent or other adult in the household often or very often … Swear at you, insult you, put you down, or humiliate you? or Act in a way that made you afraid that you might be physically hurt?”).

On the basis of the number of types of ACEs, a cumulative ACE score is calculated by adding the number of “yes” answers given on each question. This adds up to a score between 0 and 10, which is basically a severity index suggesting how many types of adversities someone has experienced in their childhood. The English version of the survey (Anda et al., 2010) was translated into Hungarian by the authors of this study. To ensure the international comparability of the results, a cross-cultural adaptation was performed with the help of an iterative forward-backward translation sequence involving an independent native speaker. Based on Felitti et al. (1998), in our current analysis cumulative scores were grouped into five categories with 0; 1; 2; 3; 4 or more ACEs. Item contents and item response options in English and Hungarian are provided in Supplementary Material.

Dube et al. (2004) found the test–retest reliability in the responses to questions about adverse childhood experiences and the resulting ACE score was found to be good and moderate to substantial.

#### 2.2.2. Strengths and Difficulties Questionnaire (SDQ)

The Strengths and Difficulties Questionnaire (SDQ) was developed for the assessment of social, emotional, and behavioral symptoms among 4–16-year-old children (Goodman et al., 1998; Turi et al., 2011). In our study the self-reported version of SDQ was used. The 25 items of the questionnaire can be grouped along 5 subscale, namely hyperactivity, emotional symptoms, conduct problems, peer relationship problems, and prosocial behavior. Each question required an answer on a three-point scale (not true = 0 points, somewhat true = 1 point, true = 2 points). First, a total score was calculated for each subscale (up to 10 points); then the totals of the subscales of peer relationship problems, emotional symptoms, conduct problems, and hyperactivity were added to get the total difficulties score (40 points at most). Higher scores suggest a higher severity of symptoms in adolescents. The Hungarian version of the Strengths and Difficulties Questionnaire was adapted and validated by Birkás et al. (2008), the questionnaire had acceptable internal consistency in the sample (Cronbach’s alpha = 0.72).

#### 2.2.3. HBSC Symptom Checklist (HBSC-SCL)

Data were collected using relevant items from the Hungarian questionnaire (Németh and Költő, 2014) of the Health Behavior in School-aged Children (HBSC) study of 2013/2014 (Inchley et al., 2016). The HBSC survey is a comprehensive measure of health behavior in school-aged children, conducted every 4 years in more than 40 countries in international cooperation with the World Health Organization, using a standardized methodology (Currie et al., 2009). The HBSC survey has been carried out in Hungary since 1985. The version currently used by Currie et al. (2009) is continuously enhanced and validated every 4 years.

Our present study included only questions related to subjective health complaints (SHC) (or psychosomatic health complaints). Adolescents were inquired about the occurrence of eight SHC: psychic and somatic symptoms including headache, stomachache, backache, feeling low, irritability, nervousness, sleeping difficulties, dizziness; and could reply with one of the following answers: almost every day/multiple times a week/about once a week/about once a month/rarely or never. Later, the frequency categories of HBSC-SCL were changed into a binary variable (multiple times a week vs. weekly or less frequently) to reduce the number of categories.

### 2.3. Statistical analysis

Statistical analyses were performed using IBM SPSS Statistics version 23.0 (IBM, Armonk, NY, United States). First, demographic data and ACE characteristics, then the mean and standard deviation of social, emotional, and behavioral (SEB) symptom scores and frequencies of SHC were described. Further examination of the psychometric properties of the ACE-10 focused on dimensionality, internal consistency, intercorrelation, internal validity analyses and association analyses for concurrent criterion validity. Dimensionality was evaluated with the help of principal component analysis. Internal consistency was measured using theta coefficient based on principal component analysis (Ercan et al., 2007) and Cronbach’s alpha, while intercorrelations, and the strength of association between two dichotomous items were evaluated with the help of Phi correlation. Point-biserial correlations were computed to study internal validity, and examine the strength of correlations between all dichotomous items and the ordinal outcome variable. To test the concurrent criterion validity, correlations between ACE accumulation and SEB symptoms were analyzed using generalized linear models. The associations between ACE accumulation and SHC were assessed using logistic regression models with entry method. All models were adjusted for age, gender, location, and maternal education. Post-test analysis was carried out using the adjusted Wald test. To determine the significance of the relationships thus identified, we have considered the recommendation of Chen et al. (2010). Chen et al. suggest that ORs, which are often difficult to interpret, should be converted into equivalents that can be matched by Cohen’s d coefficient. Their suggestion is that OR less than 1.5 is equivalent to <0.2 d, and OR > 5 is equivalent to 0.8 d.

## 3. Results

### 3.1. Descriptive statistics of the samples

The total sample consisted of 792 adolescents (59.3%) girls, mean age 14.98 years (*SD* = 1.19). Of them 52.8% (*n* = 418) reported no ACE, while 21.7% (*n* = 172) reported one, 11.1% (*n* = 88) reported two, 4.7% (*n* = 37) reported three, and 6.2% (*n* = 49) reported four or more ACEs. The most frequent type of reported child maltreatment was emotional neglect (13.6%, *n* = 108) and emotional abuse (13.4%, *n* = 106). The least prevalent reported child maltreatment was psychical neglect (2.8%, *n* = 22). Parental divorce or separation (24.7%, *n* = 196), followed by household substance abuse (8.3%, *n* = 66), were the most prevalent reported dysfunctional household condition, while the least prevalent was household physical violence (3.5%, *n* = 28). Between the total sample and the subsample no statistically significant differences emerged regarding demographic and ACE characteristics (Pearson’s chi-squared test) (Table 1).

As regards gender, the Pearson’s chi-squared test indicated that the accumulation of adversities is significantly more frequent among girls: 8.55% reported four or more adversities, and 27.41% reported two or more adverse experiences. Moreover, almost twice as many girls as boys reported three adversities. Among girls, emotional neglect (19.30%, *n* = 88) and emotional abuse (16.22%, *n* = 74) were the most prevalent reported maltreatments and parental separation/divorce (26.54%, *n* = 121) was the most common family dysfunction. Among boys, emotional abuse (9.09%, *n* = 28) and physical abuse (6.49%, *n* = 20) were the most frequent forms of maltreatment and the most prevalent reported household dysfunction was parental separation/divorce (23.05%, *n* = 121). As regards the reported prevalence of adverse childhood experiences by genders, we found a significant difference in four cases. Emotional abuse, emotional neglect, household mental illness and household substance abuse were more prevalent in girls. Table 2 presents the prevalence of adverse childhood experiences in the sample, overall and by gender.

**Table 2:** Prevalence of adverse childhood experiences (ACEs) in the sample, overall and by gender.

	Boys *n* = 308	Girls *n* = 456	Total sample *n* = 764	ꭓ^2^	*p*-value
ACE score n (%)					
0	188 (61.04)	203 (44.52)	418 (54.71)		
1	71 (23.05)	101 (22.15)	172 (23.01)		
2	29 (9.42)	59 (12.94)	88 (11.52)	16.61^†^	0.002
3	10 (3.25)	27 (5.92)	37 (4.84)		
≥4	10 (3.25)	39 (8.55)	49 (6.41)		
ACE categories exposure n (%)				ꭓ^2^ (df=9)	*p*-value
Emotional abuse	28 (9.09)	74 (16.22)	176 (20.04)	8.10	0.004
Physical abuse	20 (6.49)	33 (7.24)	53 (6.94)	0.16	0.692
Sexual abuse	8 (2.60)	21 (4.61)	29 (3.80)	2.03	0.154
Emotional neglect	15 (4.87)	88 (19.30)	103 (13.48)	32.81	<0.001
Physical neglect	7 (2.3)	14 (3.07)	21 (2.75)	0.44	0.508
Parental separation/divorce	71 (23.05)	121 (26.54)	192 (25.13)	1.19	0.276
Witnessing violent treatment of mother	9 (2.92)	19 (4.17)	28 (3.66)	0.81	0.369
Household substance abuse	17 (5.52)	46 (10.09)	63 (8.25)	5.07	0.024
Household mental illness	12 (3.90)	49 (10.75)	61 (7.98)	11.74	<0.001
Incarcerated household member	17 (5.52)	24 (5.26)	41 (5.37)	0.02	0.877

Pearson’s chi-squared test, df = 1; ^†^df = 4.

Table 3 presents the descriptive statistics of SEB symptoms and SHC in the subsample. The mean scores of SEB symptoms ranged from 2.05 (*SD* = 1.58) (peer relationship problems) to 3.6 (*SD* = 1.99) (hyperactivity/inattention problems), while the mean for the total difficulties score was 10.59 (*SD* = 5.05). In terms of SHC, more than one third of the respondents (37.5%, *n* = 254) reported nervousness, around one quarter said they were feeling low (28.1%, *n* = 190) and suffered from headache (24.8%, *n* = 168) multiple times a week and had sleeping difficulties (22.6%, *n* = 153). The prevalence of the other SHC ranged from 12 to 19.1% in the sample.

**Table 3:** Social, emotional, and behavioral (SEB) symptoms, subjective health complaints (SHC) characteristics of the subsample.

	Subsample *n* = 677
SEB symptoms mean (SD)	
Emotional symptoms	2.81 (2.44)
Conduct problems	2.13 (1.43)
Hyperactivity/inattention problems	3.6 (1.99)
Peer relationship problems	2.05 (1.58)
Total difficulties	10.59 (5.05)
SHC n (%) (multiple times a week)	
Headache	168 (24.81)
Stomachache	98 (14.47)
Backache	115 (16.98)
Feeling low	190 (28.06)
Irritability	129 (19.05)
Nervousness	254 (37.51)
Sleeping difficulties	153 (22.59)
Dizziness	81 (11.96)

### 3.2. Dimensionality, internal consistency, and intercorrelations

The dimensionality of the questionnaire was tested with the help of principal component analysis (Table 4). The results of the analysis show a relatively small total variance (44.88%) and the analysis supports the unidimensional structure of the questionnaire. The Cronbach’s alpha reliability score of the ACE-10 was proved to be equal to 0.64, however, the theta reliability coefficient based on the component analysis indicates appropriate consistency of the unidimensional model (theta = 0.86).

**Table 4:** Results of the principal component analysis.

PC	Eigenvalues	% of variance	Cumulative %
1	4.49	44.88	44.88
2	1.34	13.38	58.26
3	0.99	9.86	68.12
4	0.90	9.03	77.15
5	0.74	7.39	84.54
6	0.47	4.76	89.31
7	0.41	4.08	93.38
8	0.38	7.76	97.15
9	0.19	1.92	99.07
10	0.09	0.93	100
Items	Weights		
ACE 1	0.89		
ACE 2	0.75		
ACE 3	0.52		
ACE 4	0.73		
ACE 5	0.79		
ACE 6	0.47		
ACE 7	0.74		
ACE 8	0.61		
ACE 9	0.69		
ACE 10	0.33		

Intercorrelations of ACEs were computed to evaluate the strength of associations between the frequency of occurrence of each adverse event. The item “Incarcerated household member” was the only ACE that barely correlated with the other types of adverse events (Table 5).

**Table 5:** Intercorrelations of adverse childhood experiences (ACEs).

	Emotional abuse	Physical abuse	Sexual abuse	Emotional neglect	Physical neglect	Parental separation/divorce	Household physical violence	Household substance abuse	Household mental illness	Incarcerated household member
Emotional abuse	–									
Physical abuse	0.44^***^	–								
Sexual abuse	0.16^***^	0.13^***^	–							
Emotional neglect	0.44^***^	0.24^***^	0.06	–						
Physical neglect	0.23^***^	0.17^***^	0.13^***^	0.20^***^	–					
Parental separation/divorce	0.13^***^	0.11^*^	0.11^**^	0.18^***^	0.13^***^	–				
Household physical violence	0.27^***^	0.22^***^	0.07^*^	0.12^***^	0.26^***^	0.13^***^	–			
Household substance abuse	0.24^***^	0.12^***^	−0.01	0.16^***^	0.17^***^	0.11^**^	0.16^***^	–		
Household mental illness	0.29^***^	0.15^***^	0.22^***^	0.26^***^	0.13^***^	0.10^**^	0.12^***^	0.14^***^	–	
Incarcerated household member	0.06	0.03	0.01	0.02	0.07	−0.00	0.08^*^	0.20^***^	0.10^***^	–

Phi correlation; ^*^p < 0.05; ^**^p < 0.01; ^***^p < 0.001.

### 3.3. Internal validity

To verify internal validity, we studied the correlations between the ACE cumulative scores and each adverse event. As presented in Table 6, at least moderate correlations suggesting the appropriate internal validity of the test were found. The lowest correlation coefficient was found for the item of incarcerated household member, while emotional abuse and emotional neglect showed the strongest correlation with the cumulative ACE score.

**Table 6:** Internal validity of the Adverse Childhood Experiences Questionnaire 10 Item Version (ACE-10) on the studied adolescent sample.

	ACE cumulative score^i^
Emotional abuse	0.70^***^
Physical abuse	0.53^***^
Sexual abuse	0.33^***^
Emotional neglect	0.61^***^
Physical neglect	0.43^***^
Parental separation/divorce	0.51^***^
Household physical violence	0.44^***^
Household substance abuse	0.47^***^
Household mental illness	0.51^***^
Incarcerated household member	0.28^***^

^i^Point-biserial correlation coefficients. ***p < 0.001.

### 3.4. Concurrent criterion validity

Generalized linear models were performed to analyze the associations of ACE accumulation and SEB symptoms adjusted for age, gender, location, and maternal education; all of which proved to be significant (*p* < 0.001). Table 7 shows that compared to reporting no ACEs, reporting two, three, and four or more ACEs was significantly associated with more overall difficulties. This relationship was strong and cumulative. When we modeled the associations between the cumulative ACE score and social, emotional, and behavioral symptoms separately, similar associations with lower B coefficients were found.

**Table 7:** Associations between exposure to and accumulation of adverse childhood experiences (ACEs) and social, emotional, and behavioral (SEB) symptoms separately and overall.

	Emotional symptoms	Conduct problems	Hyperactivity/inattention problems	Peer problems	Total difficulties
	B (95% CI)	B (95% CI)	B (95% CI)	B (95% CI)	B (95% CI)
ACE score (ref.: 0)					
1	0.23 (−0.22–0.66)	0.28 (0.20–0.54)*	−0.17 (−0.39–0.35)	0.04 (−0.25–0.33)	0.53 (−0.36–1.42)
2	1.43 (0.86–1.99)***	0.82 (0.49–1.14)***	0.82 (0.36–1.28)***	0.69 (0.33–1.05)***	3.375 (2.66–4.86)***
3	1.22 (0.37–2.07)**	1.12 (0.62–1.63)***	1.53 (0.82–2.24)***	1.25 (0.70–1.81)***	5.13 (3.42–6.84)***
4 or more	1.86 (1.12–2.61)***	1.22 (0.77–1.66)***	1.59 (0.96–2.21)***	1.26 (0.77–1.75)***	5.93 (4.43–7.43)***
Age	0.07 (−0.09–0.22)	0.05 (−0.41–0.14)	0.04 (−0.08–0.171)	0.17 (0.07–0.27)**	0.33 (0.26–0.64)*
Gender (ref.: Boys)					
Girls	0.92 (0.56–1.28)***	−0.24 (−0.45 to −0.02)*	−0.04 (−0.34–0.27)	0.06 (−0.18–0.29)	0.71 (−0.16–1.43)
Location (ref.: Village)					
Town	0.06 (−0.13–0.85)	0.01 (−0.25–0.27)	0.21 (−0.15–0.58)	0.02 (−0.27–0.31)	0.30 (−0.57–1.18)
City	0.36 (−0.38–0.49)	0.06 (−0.24–0.35)	0.05 (−0.36–0.46)	−0.09 (−0.41–0.23)	0.37 (−0.61–1.36)
Maternal education (ref.: Primary)					
Secondary	−0.09 (−0.73–0.56)	0.37 (−0.75–0.13)	−0.13 (−0.66–0.42)	0.33 (−0.09–0.75)	−0.25 (−1.55–1.04)
Tertiary	0.10 (−0.55–0.74)	−0.31 (−0.69–0.76)	−0.15 (−0.69–0.39)	−0.04 (−0.46–0.38)	−0.40 (−1.70–0.90)

Generalized linear models, adjusted for age, gender, location, and maternal education.

*p < 0.05; **p < 0.01; ***p < 0.001.

We used logistic regressions of ACE with SHC adjusted for age, gender, location, and maternal education. All models were significant (*p* < 0.001). Table 8 demonstrates that ACE accumulation significantly predicted several times higher odds for experiencing each measured SHC multiple times a week. Compared to no childhood exposure, the odds ratio for feeling backache multiple times a week increased by 1.8 times after reporting one ACE. Exposure to two ACEs increased the odds of reporting an SHC from 2.11 to 3.375 times higher, while three ACEs from by 3.43 to 9.70 times, and four ACEs from by 2.43 to 6.82 times higher. These results are also reflected in the Cohen’s d effect size equivalents.

**Table 8:** Associations between exposure to and accumulation of adverse childhood experiences (ACEs) and subjective health complaints (SHC).

	Headache		Stomachache		Backache		Feeling low	
	OR (95% CI)	Effect sizes (d)^†^	OR (95% CI)	Effect sizes (d)^†^	OR (95% CI)	Effect sizes (d)^†^	OR (95% CI)	Effect sizes (d)^†^
ACE score (ref.: 0)								
1	1.13 (0.70–1.84)	<0.2	1.18 (0.62–2.26)	<0.2	1.80 (1.06–3.05)*	>0.2 and < 0.8	1.41 (0.88–2.25)	<0.2
2	2.11(1.24–3.60)**	>0.2 and < 0.8	2.30 (1.19–4.44)*	>0.2 and < 0.8	1.70 (0.88–3.25)	>0.2 and < 0.8	3.38 (2.01–5.70)***	>0.2 and < 0.8
3	3.51(1.61–7.63)**	>0.2 and < 0.8	7.25 (3.15–16.68)***	> 0.8	6.79 (2.99–15.41)***	> 0.8	5.90 (2.67–13.04)***	> 0.8
4 or more	3.13 (1.56–6.26)**	>0.2 and < 0.8	4.88 (2.28–10.42)***	>0.2 and < 0.8	2.75(1.26–5.88)**	>0.2 and < 0.8	6.82 (3.32–14.02)***	> 0.8
Age	1.15 (0.98–1.34)	<0.2	1.30 (1.05–1.6)*	<0.2	1.31 (1.14–1.66)**	<0.2	1.17 (1.00–1.37)*	<0.2
Gender (ref.: Boys)								
Girls	0.49 (0.33–0.74)**	<0.2	3.21 (1.81–5.72)***	>0.2 and < 0.8	1.95 (1.21–3.12)**	>0.2 and < 0.8	2.25 (1.51–3.36)***	>0.2 and < 0.8
Location (ref.: Village)								
Town	0.97 (0.577–1.64)	<0.2	1.49 (0.80–2.76)	< 0.2	0.78 (0.46–1.33)	< 0.2	0.98 (0.61–1.59)	<0.2
City	0.91 (0.59–1.41)	<0.2	1.01 (0.54–2.15)	< 0.2	0.60 (0.33–1.09)	< 0.2	1.39 (0.83–2.33)	<0.2
Maternal education (ref.: Primary)								
Secondary	1.35 (0.73–2.53)	<0.2	0.46 (0.21–0.97)*	<0.2	1.08 (0.49–2.38)	<0.2	0.79 (0.41–1.52)	<0.2
Tertiary	0.54 (0.35–0.83)**	<0.2	0.66 (0.31–1.39)	<0.2	1.74 (0.79–3.82)	>0.2 and < 0.8	0.94 (0.49–1.81)	<0.2

	Irritability		Nervousness		Sleeping difficulties		Dizziness	
	OR (95% CI)	Effect sizes (d)^†^	OR (95% CI)	Effect sizes (d)^†^	OR (95% CI)	Effect sizes (d)^†^	OR (95% CI)	Effect sizes (d)^†^
ACE score (ref.: 0)								
1	1.67 (0.98–2.82)	>0.2 and < 0.8	1.25 (0.82–1.90)	<0.2	1.18 (0.72–1.93)	<0.2	1.14 (0.58–2.23)	<0.2
2	2.96 (1.67–5.27)***	>0.2 and < 0.8	2.92 (1.76–4.83)***	>0.2 and < 0.8	2.51 (1.46–4.31)**	>0.2 and < 0.8	2.25 (1.13–4.46)*	>0.2 and < 0.8
3	4.90 (2.19–10.98)***	>0.2 and < 0.8	9.70 (3.79–24.77)***	>0.8	3.43 (1.56–7.51)**	>0.2 and < 0.8	1.63 (0.56–4.72)	>0.2 and < 0.8
4 or more	4.27 (2.06–8.84)***	>0.2 and < 0.8	2.73 (1.38–5.39)**	>0.2 and < 0.8	3.65 (1.82–7.30)***	>0.2 and < 0.8	5.68 (2.63–12.24)***	>0.8
Age (years)	1.16 (0.97–1.38)	< 0.2	1.25 (1.08–1.45)**	<0.2	1.30 (1.07–1.55)**	<0.2	1.15 (0.92–1.42)	
Gender (ref.: Boys)								
Girls	1.77 (1.13–2.78)*	>0.2 and < 0.8	1.72 (1.21–2.45)**	>0.2 and < 0.8	1.27 (0.85–1.87)	<0.2	2.08 (1.17–3.68)*	>0.2 and < 0.8
Location (ref.: Village)								
Town	1.57 (0.89–2.76)	>0.2 and < 0.8	1.29 (0.83–1.96)	<0.2	1.07 (0.67–1.72)	<0.2	1.67 (0.86–3.24)	>0.2 and < 0.8
City	1.88 (1.03–3.45)*	>0.2 and < 0.8	1.35 (0.83–2.17)	<0.2	0.75 (0.44–1.30)	<0.2	1.60 (0.76–3.33)	>0.2 and < 0.8
Maternal education (ref.: Primary)								
Secondary	0.54 (0.27–1.08)	< 0.2	0.51 (0.27–0.95)*	< 0.2	0.76 (0.40–1.46)	<0.2	0.41 (0.19–0.86)*	<0.2
Tertiary	0.77 (0.39–1.54)	< 0.2	0.82 (0.45–1.51)	< 0.2	0.98 (0.51–1.89)	<0.2	0.36 (0.17–0.78)**	<0.2

Logistic regression with entry method, adjusted for age, gender, location, and maternal education. *p < 0.05; **p < 0.01; ***p < 0.001.

† Cohen’s d equivalents based on Chen et al., 2010; OR < 1.5 → Cohen’s *d* < 0.2; OR > 5 → Cohen’s *d* > 0.8.

## 4. Discussion

This paper provides a comprehensive analysis of the psychometric properties of the ACE-10 in a sample of 12–17-year-old school adolescents in Hungary. It is the first study to evaluate the dimensionality, internal consistency, criterion validity and reliability of the 10-item short form of the original ACE questionnaire, which measures 5 types of maltreatment (physical, emotional, sexual abuse, and physical and emotional neglect), and 5 types of household dysfunctions.

In children/adolescents who undergo terrifying experiences on a repetitive, sometimes daily basis without being provided support and the basic safety they would need, all aspects of personality development will be seriously affected. These children would desperately need help; this is why it is extremely important to find these children exposed to adverse experiences, and provide them with proper treatment (Sethi et al., 2013). One tool for this could be the ACE-10. In contrast to the previously validated ACE-ASF questionnaire for example (Meinck et al., 2017), the ACE-10 is able to measure not only maltreatment (abuse+neglect) but also household dysfunction, which can provide a more comprehensive picture of intrafamilial adversities children may face. With our study, we wanted to fill the gap left by the lack of validated measures to screen for adverse experiences.

### 4.1. Dimensionality, reliability, and validity

#### 4.1.1. Dimensionality, reliability, and intercorrelation

Our results suggest that the ACE-10 questionnaire can be interpreted as a unidimensional construct. The assessment tool showed appropriate internal consistency and reliability having a theta coefficient of 0.86 and a Cronbach’s alpha of 0.64. It is important to point out here, that the theta coefficient is a more reliable indicator of the unidimensional construct of the ACE-10 based on principal component analysis (Armor, 1973; Ercan et al., 2007). The psychometric properties of the ACE-10, investigated in our present study, have only been examined in a German study so far (Wingenfeld et al., 2011). The sample of the German study had three subsamples: inpatients of a clinic for psychosomatics and psychotherapy, a subsample of students, and a subsample of adults from the general population. Wingenfeld and colleagues proved a good internal consistency of the questionnaire (Cronbach’s alpha = 0.76). The target population in their study consisted of adult patients with mental disorders. We consider this important to highlight in terms of the alpha consistency of the questionnaire because—as the literature and our results suggest (Felitti et al., 1998; Hughes et al., 2017; Bellis et al., 2019)—the prevalence of exposure to adverse childhood experiences is higher in the population with mental disorders. We tested adolescents from a normal population, which may explain the difference between the alpha coefficients found in the two studies. Moreover, the low number of items tested may result in a lower Cronbach’s alpha coefficient (Tavakol and Dennick, 2011; Taber, 2018). Generally, internal consistency has been found to be high across studies in different cultural contexts using different versions of the ACE (Bruskas and Tessin, 2013; Kazeem, 2015; Helitzer et al., 2016).

Intercorrelation analysis shows that the particular ACE dimensions represented in the test correlated in most cases with each other in the form of low to moderate correlations. We found the highest degree of intercorrelations between emotional abuse and physical abuse, and between emotional abuse and emotional neglect. Between the dimension of incarcerated household member and the other ACEs only non-significant or significant but barely correlating relationships were found. Correlations between other items were mild or moderate. Our results are broadly in line with those of the German study. They found the least strong intercorrelations between ACEs of incarcerated household member, separation/divorce, and household mental illness and the rest of the ACEs; and the highest intercorrelations between physical and emotional abuse (Wingenfeld et al., 2011). Thus, both the study of Wingenfeld and colleagues and our study confirm that the items of the ACE-10 capture the different but interrelated domains of early adverse experiences. Previous studies have also concluded that these events represent different experiences (Scher et al., 2001; Bremner et al., 2007), even if these are aspects of harmful childhood experiences that are often cumulative (Dong et al., 2004).

The low correlations on the item of incarcerated household member in our present study could be explained by the fact that this harm was the household dysfunction with the lowest incidence in our sample. In addition, the ACE study (Felitti et al., 1998) with its sample of 17,337 respondents also found this harmful experience to be the least prevalent (4.7%) of the 10 ACEs.

#### 4.1.2. Internal validity

The items in the ACE-10 have at least a moderate correlation with the cumulative ACE score, meaning that all items in the questionnaire are relevant to the measurement of the phenomenon the questionnaire is testing. Emotional abuse (*r* = 0.70) and emotional neglect (*r* = 0.61) were found to most correlate with the ACE cumulative score. From a sample of over 18,000 people Dong et al. (2004) found that the prevalence of emotional abuse and emotional neglect is associated with the highest risk of additional harms. Their results show that a child who has experienced one type of ACE is at least 81% more likely to have experienced other adverse events as well. This means that in most cases harmful experiences are co-occurring. This explains the at least moderately strong correlation coefficients obtained in our current study.

#### 4.1.3. Concurrent criterion validity

The ACE-10 demonstrated a good concurrent criterion validity in our sample. Our hypothesized correlations of cumulative ACE score with SEB symptoms and SHC were confirmed, and these relationships were strong and mostly graded. Our results are in line with previous studies that provided evidence on the negative impact of childhood adversities on mental and subjective health in adolescence (Norman et al., 2012; Meinck et al., 2017; Rebicova et al., 2019). For backache, stomachache, and nervousness, ACE exposure was associated with more frequent symptoms, but the association was not fully graded, with three ACEs increasing the risk for morbidity more than four or more ACEs. The small sample size in the categories of three (*n* = 31) and four or more exposures (*n* = 41) makes it plausible that comparisons at these levels are less accurate and conclusions about the observed graded and non-graded relationships should be taken with caution.

#### 4.1.4. Strengths and limitations

All studies except one (Meinck et al., 2017) used adult retrospective self-report, which may make their findings prone to recall bias (Hardt and Rutter, 2004), our present study used current self-report of ACEs among youth. There is evidence that the participation of children and adolescents in such research is necessary and justified, and the obtained data are suitable for analysis (Riley, 2004; Finkelhor et al., 2016). It is also important to get schools involved in prevalence studies, which should result in more detailed and more reliable data (Mathews et al., 2020).

Like all studies, ours also has limitations. First, the ACE-10 is a short retrospective 10-item screening tool, which may lead to biased results or reported ACEs to get lost. The sensitivity of the topic may have had an impact on participants’ willingness to talk about these adverse events (Tourangeau and Yan, 2007). At the same time, adolescents’ self-report is considered to be more reliable than agency records, parental report of adolescent victimization, and adult retrospective self-report (Johnsona et al., 2002; Hardt and Rutter, 2004). Second, the present study is not quite varied, as it only included 12–17-year-old children in Hungary. Consequently, our findings cannot be applied unambiguously for other age groups, or more vulnerable populations. Third, schools and participants were not randomly selected and the inability to generalize due to the non-representative sample. Fourth we did not have the opportunity to compare the ACE-10 to other trauma and adversity measures as they lack Hungarian adaptation and validation. Fifth, our measure included an item involving several serious physical abuse types besides spanking (e.g., kick, punch or beat up). Although the harmful effects of recurring spanking have been well established (Afifi et al., 2017; Merrick et al., 2017), prior research distinguished multiple instances from none or very few instances of spanking. Spanking, therefore, should become an individual item in future research employing this measure.

### 4.2. Practical implications

The ACE-10 can be used to assess the extent of childhood adversities among adolescents, and measure changes in the victimization of maltreatment (abuse, neglect) and household dysfunctions in a time- and cost-effective way through repeated cross-sectional measures.

The advantage of the ACE-10 is that the test can be taken in a short time, and the options to the answer are simple and easy to understand. Due to its brevity, it can be included in multi-component studies or used by itself in short surveys. It might be useful to repeat the test on a larger sample and compare the data with other outputs, e.g., PTSD data.

The ACE-10 is a valuable measure for consideration in child abuse research and should be more widely used and tested. This measure fills a significant gap among measures for validated adverse childhood experiences.

Our study was conducted in adolescents, and demonstrates that the ACE-10 is suitable for assessing intrafamilial adverse childhood experiences in adolescents. Further, more research is needed to validate child abuse measures using current child self-report in adolescent samples (Mathews et al., 2020).

## 5. Conclusion

Our results confirm that the Hungarian version of the ACE-10 is a valid measure of physical, emotional, and sexual child abuse, physical and emotional neglect and household dysfunction among adolescents. Even with the limitations, there is more evidence to support the validity of the ACE-10.

It is important to identify children at risk of maltreatment and family dysfunctions as early as possible. This requires valid measurement tools to document and quantify our investigations.

By describing the characteristics of the adolescent population in this way, data can be more reliable, prevention measures can be better planned and adapted to the population. Adolescence is particularly important and one of the most critical periods of development. In terms of prevention, risk reduction and early intervention, it is one of the optimal life cycles in which preventive and therapeutic interventions can be implemented, even at school level, to achieve more effective change in the population at risk.

It is important to monitor the frequency of ACEs in order to inform child welfare agencies and policy makers. Our current research is focused on the general adolescent population, so the most vulnerable, the severely disadvantaged and those under child protection services were not included in this study; we plan, however, to validate the ACE-10 on this vulnerable population.

## Data availability statement

The raw data supporting the conclusions of this article will be made available by the authors, without undue reservation.

## Ethics statement

Ethics approval was issued by the Research Ethics Committee of the Hungarian Medical Research Council under the approval number ETT TUKEB 47848–7/2018/EKU. Written informed consent to participate in this study was provided by the participants’ legal guardian/next of kin.

## Author contributions

BK-T, BO, and ZF: conception, design of the work, data analysis, and interpretation. BK-T and BO: data collection. BK-T: Drafting the article. BK-T, BO, IS, and ZF: critical revision of the article and final approval of the version to be published. All authors contributed to the article and approved the submitted version.

## Conflict of interest

The authors declare that the research was conducted in the absence of any commercial or financial relationships that could be construed as a potential conflict of interest.

## Publisher’s note

All claims expressed in this article are solely those of the authors and do not necessarily represent those of their affiliated organizations, or those of the publisher, the editors and the reviewers. Any product that may be evaluated in this article, or claim that may be made by its manufacturer, is not guaranteed or endorsed by the publisher.

## Abbreviations

ACEs: Adverse Childhood Experiences
ACE-ASF: Adverse Childhood Experiences Abuse Short Form
HBSC: Health Behavior of School Children
HBSC-SCL: Health Behavior of School Children Symptom Checklist
SEB: Social, Emotional, and Behavioral Symptoms
SDQ: Strengths and Difficulties Questionnaire
