# Supplementary material for: Psychometric properties of the Adverse Childhood Experiences Questionnaire 10 item version (ACE-10) among Hungarian adolescents
Source: Front Psychol. 2023 May 19;14:1161620. doi: 10.3389/fpsyg.2023.1161620 (PMC10235773; doi:10.3389/fpsyg.2023.1161620)
Supplement: Supplementary file 1 [file Table_1.pdf]

Supplementary material

The ACE-10—preambles, item contents and response options

**Item**	**Preamble and Content**	**ACE Category**
	During your life:	
1[a]	Did a parent or other adult in the household often or very often ... Swear at you, insult you, put you down, or humiliate you? or Act in a way that made you afraid that you might be physically hurt?	Emotional abuse
2[a]	Did a parent or other adult in the household often or very often ... Push, grab, slap, or throw something at you? or Ever hit you so hard that you had marks or were injured?	Physical abuse
3[a]	Did an adult person at least 5 years older than you ever ... Touch or fondle you or have you touch their body in a sexual way? or Attempt or actually have oral, anal, or vaginal intercourse with you?	Sexual abuse
4[a]	Did you often or very often feel that ... No one in your family loved you or thought you were important or special? or Your family didn't look out for each other, feel close to each other, or support each other?	Emotional neglect
5[a]	Did you often or very often feel that ... You didn't have enough to eat, had to wear dirty clothes, and had no one to protect you? or Your parents were too drunk or high to take care of you or take you to the doctor if you needed it?	Physical neglect
6[a]	Were your parents ever separated or divorced?	Parental separation/divorce
7[a]	Was your mother or stepmother: Often or very often pushed, grabbed, slapped, or had something thrown at her? or Sometimes, often, or very often kicked, bitten, hit with a fist, or hit with something hard? or Ever repeatedly hit for at least a few minutes or threatened with a gun or knife?	Witnessing violent treatment of mother
8[a]	Did you live with anyone who was a problem drinker or alcoholic or who used street drugs?	Household substance abuse
9[a]	Was a household member depressed or mentally ill, or did a household member attempt suicide?	Household mental illness
10[a]	Did a household member go to prison?	Incarcerated household member

[a] Dichotomous scales–yes/no.
