# Supplementary material for: Psychometric properties of the Adverse Childhood Experiences Questionnaire 10 item version (ACE-10) among Hungarian adolescents
Source: Front Psychol. 2023 May 19;14:1161620. doi: 10.3389/fpsyg.2023.1161620 (PMC10235773; doi:10.3389/fpsyg.2023.1161620)
Supplement: Supplementary file 2 [file Table_2.pdf]

Ártalmas Gyermekkori Élmények Kérdőív (ACE-10)

Kérdőív- instrukció, itemek tartalma és válaszlehetőségek

Item	Instrukció és az itemek tartalma	ACE kategóriák
	A következő kérdések a gyermekkorodra vonatkoznak. Kérjük, hogy amennyiben az alábbi állítások bármelyikét átélted ebben az időszakban, jelöld meg!	
1[a]	Valamelyik szülő vagy más a háztartásban élő felnőtt gyakran/ vagy nagyon gyakran: szidott, sértegetett, elnyomott, megalázott? vagy Olyan módon viselkedett, hogy attól féltél, hogy fizikailag megsérülsz?	Érzelmi abúzus
2[a]	Valamelyik szülő vagy más a háztartásban élő felnőtt gyakran/ vagy nagyon gyakran: meglökött, megütött, erősen megragadott, vagy valamit hozzád vágott? vagy Valaha is ütött meg olyan erővel, hogy annak látható nyoma maradt, és Te megsérültél?	Fizikai abúzus
3[a]	Egy Tőled legalább 5 évvel idősebb személy valaha: fogdosta vagy simogatta az intim testrészeidet, vagy Neked fogdosni/simogatni kellett a másik személy intim testrészeit? vagy Megpróbált vagy megvalósított Veled orális, vagy anális, vagy vaginális közösülést?	Szexuális abúzus
4[a]	Gyakran/ vagy nagyon gyakran érezted: hogy a családodból senki nem szeret, vagy nem tart Téged különlegesnek és fontosnak? vagy A családtagjaid nem figyeltek oda egymásra, nem érezték egymáshoz közel magukat, nem támogatták egymást?	Érzelmi elhanyagolás
5[a]	Gyakran/ vagy nagyon gyakran érezted, hogy: nem volt elég ennivalód, koszos ruhákat kellett viselned, és nem volt, aki megvédjen Téged? vagy A szüleid túlságosan sokat ittak vagy drogoztak, hogy gondodat viseljék, vagy orvoshoz vigyenek, amikor szükséged volt rá?	Fizikai elhanyagolás
6[a]	Elváltak vagy különéltek a szüleid?	Szülők különélése/válása
7[a]	Az édesanyádat vagy nevelőanyádat: gyakran/ vagy nagyon gyakran meglökték, erősen megragadták, megütötték vagy valamit hozzávágtak? vagy Néha, gyakran, vagy nagyon gyakran rugdosták, harapták, ököllel vagy kemény tárggyal ütötték? vagy Valaha perceken át folyamatosan ütötték, vagy késsel, fegyverrel megfenyegették?	Anya ellen elkövetett erőszak szemtanúja
8[a]	Éltél együtt olyan személlyel, aki problémás ivó vagy alkoholista volt, vagy kábítószert használt?	Alkoholt vagy egyéb pszichoaktív szert túlzottan használó családtag

9[a]	Családodban küzdött valaki depresszióval, vagy szenvedett mentálisan betegségben, vagy kísérelt meg öngyilkosságot?	Mentális betegségben szenvedő, vagy öngyilkosságot megkísérelt családtag
10[a]	Előfordult a családodban, hogy valaki börtönbe került?	Börtönviselt családtag

[a]Dichotóm skála – igen/nem válaszok adása lehetséges
